# Practical Notes and Formulæ

**Published:** 1887-10

**Authors:** 


### PRACTICAL NOTES AND FORMULÆ.

**Ear-Ache** (Peoria Medical Monthly).—

℞. Muriate of morphia................................gr. v,
Sulphate of atropia................................gr. i,
Ol. olivæ................................ʒ i,
Neutral glycerin................................ʒ iss.

M. Sig. Drop from 3 to 5 drops in the ear, and repeat every hour until relieved from pain, taking care to plug the ear with cotton after applying the medicine.

**Gonorrhœa Treatment.**—J. B. Jones says that, as an injection for urethritis, especially the acute form, there is nothing superior to the following:

℞. Ext. opii................................gr. xx,
Glycerinæ................................fl ℥ i,
Zinci sulphatis................................gr. vi,
Aquæ destill................................fl ℥ v.

M. Sig. Inject every three hours.

As a diuretic where the urine is highly acid and the object is to increase the amount of water:

℞. Potassi acetatis................................℥ ss,

Spt. nitr. dulcis................................fl ℥ ss,

Aquæ camphoræ................................fl ℥ xv.

M. Sig. One tablespoonful every three hours.

**Iodoform in Phthisis.**—Huchard combines iodoform with creasote in the treatment of phthisis as follows:

℞. Iodoform................................
Creasote................................
Pulv. benzoin................................
Balsam tolu................................aa gr j.

M. Sig. Two to 4 pills daily.—*Journal de Medicine.*

**The Medical Treatment of Fissures in Ano by Cocaine.**—M. Mendel commences by cauterizing the affected surface with nitrate of silver after first applying a five per cent. solution of cocaine. He then orders the patient to apply, especially after defacation, the following ointment:

℞. Acidi boracici................................gr. xxx,
Cocain. hydrochlor................................gr. xv,
Lanolin ................................ʒ v.

M.

In the intervals of defacation he applies to the wound lint coated with this ointment, and prevents constipation by using saline purgatives. Moreover, he cleanses the surface of the fissure several times a day with three per cent. boric acid water. In this way Mendel obtained recovery in from one to two years.—*Le Concours Medical.*

**Diphtheria.**—A writer (in *Eclectic Medical Journal*) gives a glowing account of the following anodyne in diphtheria:

℞. Merck's volatile extract of pine needles........
Merck's resorcin..............................aa ʒ ij,
Fluid ext. pinus canadensis......................℥ j,
Glycerine..................................℥ ss,
Aqua (hot) q. s. or..............................℥ iij.

M. S. Use in the nose and throat with an inhaler or atomizer several times a day.

℞. Merck's resorcin............................℥ j, *sen* ss,
Fluid ext. pinus canadensis.....................℥ ij,
Glycerine...................................℥ ij,
Aqua (hot)..............................f ℥ iv,

M. S. Dose, half to a drachm every two or three hours.

Should the throat be very bad, a preparation twice as strong as that advised for the atomizer is to be used with a probang, and repeated three times a day. I have observed the action of the remedy in fourteen cases. One was in my own family, a boy of seven years; the results were all that could be desired.

**Parson's Local Anæsthetic.**—

℞. Chloroform...............................parts xii,
Tr. aconite............................... " xii,
Tr. capsicum.............................. " v,
Tr. pyrethrum............................. " ii,
Oil cloves................................ " ii,
Camphor................................... " ii.

Dissolve the camphor in the chloroform, then add oil of cloves, and then the tinctures.

The venerable Dr. Parsons, in sending this formula for publication, says: "I cannot expect to remain much longer in this world, and I want the profession to know the value of this local anæsthetic."—*The Southern Dental Journal.*

**Antiseptic Mouth Wash.**—

R. Sodæ biboratis........................gramme 1,
Thymol..............................gramme 0-50,
Aqua destill..........................grammes 300.

Ft. Sol.

This preparation is said to be an excellent corrective for fœtid breath, when it proceeds from decaying matter in carious teeth, etc.—*Magiot, in Gazette Hebdomadaire.—Medical Press and Circular.*

**A Formula for Lithuria.**—Dr. J. B. Johnson, in *Medical Reporter:*

℞. Liq. ammoniæ citrat.......................... ℥ jss,

Sodæ phosphatis.......... ................ ʒ jss,

Acidi salicylic............... ............... ʒ jss,

Ferri pyrophosphati.......................... ℈ ij,

Glycerini.................................. ℥ ij,

Elixir aurantii............................. ʒ vj,

Aquæ q. s. ad ...... ....................... ℥ viij.

S. Mix in the order of the formula, shake well, and give a tablespoonful every three or four hours.

I have found no prescription superior to this in cases of lithuria, and in uric acid diatheses attended with gouty and rheumatic symptoms; and under such circumstances, the mixture is much improved by the addition, to each dose, of 20 drops of wine of colchicum. In combination with drachm-doses of either of the fluid extracts of pariera brava, or dog grass, it has served me well in chronic cystitis, gonorrhœa and leuchorrœa. The fluid ext. of pichi, in suitable doses, has also, proved an excellent addition to the formula in the treatment of mucus discharges from the urinary organs.

**Duration of Infection of Eruptive Fevers.**—My observations make the duration of infection in the several diseases as follows: Measles, from the second day, for exactly three weeks. Small-pox, from the first day, under one month, probably three weeks. Scarlet fever, at about the fourth day, for six or seven weeks. Mumps, under three weeks. Diphtheria, under three weeks.—*British Medical Journal.*

**Chronic Rheumatism.**—

℞. Liq. potassi arsenitis.......................... ʒ ss,

Potassii acetatis............................ ʒ ii,

Vini colchici rad............................ ʒ ij,

Ext. cimicifugæ.......................... fl ʒ iij,

Ext. phytolacca........................... fl ʒ iss,

Aqua menth. pip.......... .................. ℥ iij.

M. Sig. Two tablespoonfuls in water every four hours.—*Ex.*

**Treatment of Chorea.**—Descroizilles prescribes as follows:

℞. Zinci valerian.......... ..................
Ext. hyoscyami...........................
Bismuth. subnitrat........................ aa gr. xv.

M. Divide in pill thirty in number.

Sig. From 3 to 6 pills daily.—*Journal de Medicine de Paris.*

